# Artificial Intelligence–Enabled Mobile Health Intervention (mDiabetes) to Reduce Diabetes Risk Behaviors in Rural India: Quasi-Experimental Pre-Post Study

**DOI:** 10.2196/79283

**Published:** 2025-12-05

**Authors:** Joshua Chadwick, Nidhi Jaswal, Janani Surya, Chandru Sivamani, Varun Ramesan, Nalini Saligram

**Affiliations:** 1 Indian Council of Medical Research National Insitute of Epidemiology Chennai, Tamil Nadu India; 2 Arogya World India Trust Bangalore, Karnataka India

**Keywords:** artificial intelligence, AI, mobile health, mHealth, AI-enabled mHealth, diabetes prevention, physical activity, rural health, community intervention, India

## Abstract

**Background:**

India faces a dual burden of diabetes and prediabetes. Although mobile health (mHealth) interventions have shown promise in promoting healthy lifestyle changes, most interventions deploy generic, “one-size-fits-all” messages that do not consider individual behavioral patterns, motivational states, or changing needs over time.

**Objective:**

This formative evaluation study aimed to assess the effectiveness of an artificial intelligence (AI)–enabled, personalized mHealth messaging intervention (mDiabetes) compared to traditional, nonpersonalized mHealth messaging in promoting engagement with diabetes risk reduction behaviors among adults in Gulbarga, Karnataka, South India.

**Methods:**

A quasi-experimental pre-post study was conducted among adults without diabetes (N=1048). Participants were divided into intervention and control groups. The control group received static diabetes prevention messages via WhatsApp, while the intervention group received customized messages twice a week based on individual feedback through reinforcement learning algorithms. Data on demographics, diabetes knowledge, and lifestyle behaviors were collected via home interviews. Chi-square tests and *t* tests were performed to assess group differences. Intervention effects were evaluated using multivariable logistic regression for binary outcomes and ANCOVA for continuous outcomes. Adjusted odds ratios (aORs) with 95% CIs were reported, and Bonferroni correction was applied for multiple comparisons.

**Results:**

A total of 1048 (96.9%) participants (n=661, 63.1%, female) completed the 6-month follow-up. At endline, no significant between-group differences were observed for primary outcomes. Both groups had similar odds of meeting the physical activity goal (≥30 minutes/day) at endline (aOR 1.0, 95% CI 0.7-1.3, *P*=.74). Baseline activity (aOR 2.1, 95% CI 1.5-3.1, *P*<.001) and age >50 years (aOR 3.8, 95% CI 1.6-9.3, *P*=.003) were significant predictors of endline physical activity, while employment was associated with lower odds of physical activity (aOR 0.2, 95% CI 0.1-0.3, *P*<.001). Daily fruit intake was modestly higher in the intervention group (aOR 1.4, 95% CI 0.8-2.3, *P*=.24), and participants aged 26-35 years had higher odds of daily fruit intake (aOR 4.7, 95% CI 1.9-11.8, *P*=.001), while employment was associated with lower odds (aOR 0.3, 95% CI 0.1-0.8, *P*=.02). The mean BMI difference at endline was –0.0 kg/m² (95% CI –0.6 to 0.5, *P*=.95), and baseline BMI was a strong predictor of endline BMI (*P*<.001). Exploratory behavioral outcomes revealed no significant differences: stair use (aOR 0.9, 95% CI 0.7-1.4, *P*=.79), walking for chores (aOR 2.4, 95% CI 1.0-6.1, *P*=.06), helping with household chores (aOR 1.0, 95% CI 0.4-2.3, *P*=.94), and farm work (aOR 1.3, 95% CI 0.9-1.8, *P*=.19).

**Conclusions:**

Both AI-enabled and traditional mHealth interventions have similar effectiveness in promoting diabetes prevention behaviors in rural India. Simple, well-designed mHealth interventions delivered through an accessible platform like WhatsApp can achieve meaningful behavior change without the need for complex AI technology. The comparable effectiveness suggests the potential for scalable, cost-effective, equitable diabetes prevention strategies in resource-limited settings.

## Introduction

Diabetes represents one of the greatest public health challenges of the 21st century, contributing to morbidity, mortality, and economic loss worldwide. Currently, 589 million adults (aged 20-79 years) worldwide have diabetes (approximately 1 in 9 adults), and this number was projected to rise to 853 million by 2025 [1,2]. India accounts for a rapidly growing disease burden, particularly among its rural and underserved populations [3]. Escalating rates of type 2 diabetes and prediabetes are closely linked to shifts in lifestyle, dietary patterns, and limited access to preventive health care [4]. Rural communities are often disproportionately impacted due to limited health care access, low health literacy, and barriers to effective behavioral risk reduction [5].

Mobile health (mHealth) interventions have emerged as promising tools for addressing such challenges by delivering targeted health education and behavior change messages directly through mobile device. A growing body of work also demonstrates that mHealth interventions, including SMS- and app-based educational programs, can improve diabetes-related health behaviors and clinical outcomes in low-resource settings [6]. Recent systematic reviews and trials show significant benefits for glycemic control, adherence, and lifestyle change in both urban and rural Indian populations [5,7]. At the same time, studies from 2024-2025 emphasize the emerging utility of artificial intelligence (AI)–powered platforms that dynamically tailor content using real-time user data, potentially setting a new standard in digital health communication [6]. Several contemporary randomized trials and reviews report that both traditional and AI-enabled mHealth modalities are feasible and beneficial, with limited but rapidly increasing direct comparisons in rural and underserved populations [8].

Despite this progress, most previous interventions deploy generic, “one-size-fits-all” messages that do not consider individual behavioral patterns, motivational states, or changing needs over time [9]. This lack of personalization may lead to low engagement and suboptimal outcomes, and few published studies have directly contrasted static mHealth with AI-personalized approaches in rural Indian settings [5].

Key uncertainties persist regarding the comparative effectiveness, user acceptability, and scalability of AI-driven personalized navigation versus simpler, static messaging strategies in real-world rural communities with varying digital access and literacy [10]. There is a pressing need to evaluate which digital strategies offer the maximum benefit for the least cost and the highest reach.

In this study, we rigorously compared, in a large rural cohort, an adaptive AI-driven mHealth intervention (dynamic) with a standard, static messaging system for diabetes prevention. To bridge the gap between the two types of interventions, this study leveraged reinforcement learning algorithms to optimize message delivery by sending customized messages based on an individual’s preferences (healthy food intake, unhealthy dietary habits, physical activity, diabetes symptom knowledge, and awareness of complications), aimed at promoting a healthy lifestyle based on the transtheoretical model of behavior change [11]. This individualized, data-driven communication framework represents an innovative strategy for enhancing engagement, contextual relevance, and behavioral adoption in diabetes prevention among rural populations. Our study will offer a model for future user-centered digital health interventions that can be customized for diverse behavioral domains beyond diabetes prevention. Therefore, this formative evaluation aimed to assess the effectiveness of an AI-enabled, personalized mHealth messaging intervention compared to traditional, nonpersonalized mHealth messaging in promoting engagement with diabetes risk reduction behaviors among adults in the rural district of Gulbarga, Karnataka, South India, while also examining how the intervention functions in real-world settings, what the participant engagement patterns are, and what the potential areas for improvement are [12].

## Methods

### Study Design, Setting, and Population

The study used a quasi-experimental pre-post design with a control group and was conducted from January to November 2022, with recruitment and baseline data collection occurring between January and March 2022 and follow-up data collection completed from September to November 2022, among the rural population in the district of Gulbarga, Karnataka, South India. Gulbarga (also known as Kalaburagi), is situated in northeastern Karnataka, part of the Deccan Plateau, and is characterized by a semiarid climate and a predominantly agrarian economy. With a population of approximately 2.5 million (based on the 2011 Census, India), the district has a literacy rate of 64.8% [13]. Mobile phone usage among the rural population has experienced significant growth in recent years, with increasing adoption for communication, agricultural information access, and digital payments, although challenges in network connectivity persist in remote areas. The study population consisted of adults without diabetes aged 18-60 years.

### Sample Size

Sample size calculations were based on a two-sided significance level of 95% and a statistical power of 80%, assuming a 10% difference in the primary outcome (physical activity) between the AI-enabled mHealth (intervention) group and the traditional mHealth (control) group. Based on these parameters, the calculated sample size was 415 participants for each group. To account for an anticipated 20% nonresponse rate, the target sample size was adjusted to 520 participants per group, resulting in a total target enrolment of 1040 participants.

### Recruitment of Participants

Frontline workers (FLWs) facilitated the recruitment through community mobilization events in Gulbarga, aimed at raising awareness about the benefits of the AI-enabled, personalized mHealth messaging intervention used in this study. All participants received comprehensive information about the study’s purpose, objectives, potential risks, and benefits before they provided written consent for participation based on the eligibility criteria. Participants’ eligibility was determined based on self-reported or absence of physician-diagnosed diabetes (convenience sampling). Individuals with a known diagnosis of diabetes mellitus or that confirmed by medical records or current use of antidiabetic medication were excluded. This study followed a quasi-experimental design, without randomization. Eligible participants were divided into two groups, intervention and control, with 541 participants in each group.

Following recruitment, participants received an opt-in link, and FLWs guided them through the WhatsApp opt-in process. Upon successful opt-in, participants began receiving diabetes prevention messages starting the next day. The first follow-up was conducted 6 months after baseline data collection, using in-person interviews to fill out the survey at follow-up, facilitated by the FLWs. Blinding was not possible due to the nature of the intervention.

### Intervention Design and Implementation

Arogya World’s mDiabetes program forms the foundation of the mHealth initiative evaluated in this study [12]. The mDiabetes program delivers diabetes prevention and control information directly to individuals’ mobile phones regardless of their risk status. Developed in 2011 in collaboration with the Rollins School of Public Health at Emory University, the standard program consists of 57 messages crafted using the transtheoretical model of behavior change. The system was built on a reinforcement learning framework using a Deep Q-Learning (DQN) algorithm, adapted for real-world mHealth deployment without prior training data, as described by Kinsey et al [14]. At baseline, participants completed a questionnaire assessing five behavioral domains: healthy food intake, unhealthy dietary habits, physical activity, diabetes symptom knowledge, and awareness of complications. Their responses were used in a warm-up phase to initialize state scores, ensuring that participants with lower baseline scores in a given domain received more relevant messages. Each week, the reinforcement learning agent delivered two customized health messages and two follow-up questions. Participant responses were used to update their individual state scores. A reward signal was generated when a participant’s score improved, and this was used to update the replay buffer.

The DQN was optimized weekly using minibatch gradient descent and a target network, with an ϵ-greedy policy applied to balance exploration and exploitation. Over time, this iterative process enabled the system to adaptively tailor content to participant needs (eg, used in the intervention arm: “You can reduce your risk of diabetes by walking briskly for 30 minutes daily; try walking to the temple or shops.”). These messages, available in 12 languages, were validated by Arogya World’s Behaviour Change Task Force comprising experts in diabetes, public health, and behavior change from both national and international spheres. Messages in the mDiabetes program are typically distributed as SMS texts, automated voice calls, or WhatsApp messages, sent twice weekly over a 6-month period. The program has reached approximately 2 million people to date, with this study using WhatsApp as the delivery platform.

**Figure 1:**
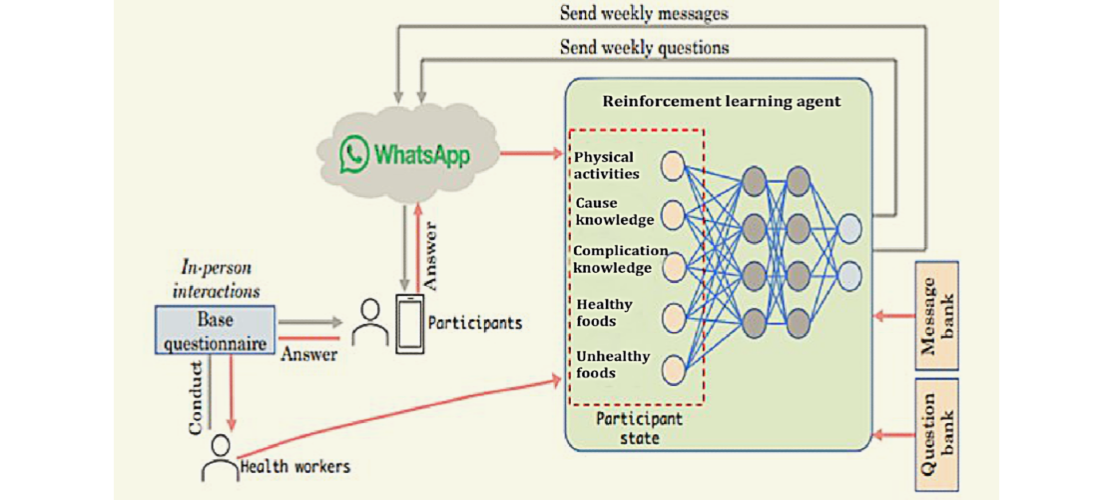
Personalized message–based intervention system overview.

A detailed description of building a customized messaging system for health intervention in underprivileged regions using reinforcement learning has been provided elsewhere (Figure 1) [14].

The mDiabetes program includes an AI-based system to enhance traditional mHealth (diabetes) interventions by developing dynamic, customized text messages to improve adherence to diabetes prevention behaviors:

- AI-enabled mHealth: This intervention was different from the traditional mHealth program due to the addition of an AI system to develop a dynamic and customized text messaging intervention based on end-user feedback. Participants in the intervention group received two customized health-related messages on WhatsApp (containing information about diabetes complications and the impact of nutrition and physical activity on diabetes prevention), coupled with two questions probing their risk profile/behavior. The subsequent week’s messages for each participant in the intervention group were based on their responses to the two lifestyle-related questions from the previous week.
- Traditional mHealth: A total of 57 static mHealth messages were delivered twice a week via WhatsApp as per a standard scheduler for a period of 6 months, focusing on improving knowledge, attitudes, and practices related to diabetes prevention behaviors, including physical activity and dietary habits [12].

### Data Collection

We assessed 2096 individuals for eligibility, of whom 1014 (48.4%) were excluded (n=598, 59%, did not meet the inclusion criteria and 416, 41%, declined participation). A total of 1082 (51.6%) participants were divided into two groups with an equal number of participants, an intervention group (n=541, 50%) and a control group (n=541, 50%). In the control group, 34 (6.3%) participants did not complete the WhatsApp opt-in process and therefore never received the intervention messages; these individuals did not contribute intervention or outcome data and were excluded from the analysis. Data for this study were collected from primary sources using structured questionnaires, direct interviews, and anthropometric measurements conducted by trained FLWs. Participant demographics, such as age, sex, education, and employment status, were recorded.

Physical activity was assessed through self-report questions on frequency and duration (≥30 minutes/day considered active), adapted from the World Health Organization guidelines on physical activity and sedentary behavior for adults [15].

Dietary habits were measured by frequency of fruit and vegetable intake per day or per week and avoidance of high-fat foods. Secondary outcomes included knowledge of diabetes symptoms, complications, and preventive behaviors. These questionnaire domains underwent expert review by public health and behavioral science specialists and were pretested in a pilot sample for contextual relevance, clarity, and cultural appropriateness before field implementation. Anthropometric measurements, including height and weight, were obtained using calibrated instruments (a SECA 213 portable stadiometer and a SECA 803 digital scale, respectively). In addition, the BMI was calculated (kg/m²), and participants were categorized as underweight (<18.5 kg/m²), normal (18.5-24.9 kg/m²), and overweight/obese (≥25 kg/m²) [16]. All responses were self-reported except for anthropometric measurements. Awareness-related items (eg, “Are you aware of diabetes?”) and knowledge questions (eg, causes, complications) were scored dichotomously (yes/no). Lifestyle questions on diet and physical activity used frequency scales (eg, daily, 3-4 times/week, rarely) The same questionnaire was administered at baseline and endline to capture change over time (see Multimedia Appendix 1). Engagement and response data from WhatsApp-delivered messages were automatically logged by the mHealth platform.

### Statistical Analysis

All statistical analyses were performed using StataMP 64 software (version 17.0). Descriptive statistics summarized the baseline characteristics and outcome variables for both groups. Categorical variables were reported as frequencies and percentages, and continuous variables were summarized using means (SDs).

Normality of continuous variables was assessed using the Shapiro-Wilk test. For normally distributed continuous variables (eg, BMI), independent-sample *t* tests were performed to compare group means. For categorical variables (eg, physical activity, dietary behaviors), chi-square tests were performed to examine group differences at both baseline and endline.

To evaluate the effect of the intervention, separate multivariable logistic regression models were constructed for each binary outcome variable (≥30 minutes of daily physical activity, daily fruit intake). Each model included the intervention group (AI-enabled vs traditional mHealth), the baseline value of the respective outcome, and additional covariates identified through univariate analysis (variables with *P*<.20 were considered for inclusion). Key demographic factors, such as age, sex, and employment status, were also retained, where appropriate. Both unadjusted odds ratios (ORs) and adjusted odds ratios (aORs) with corresponding 95% CIs were reported. For continuous outcomes, such as the BMI, ANCOVA was performed, with endline BMI as the dependent variable and baseline BMI included as a covariate to control for initial differences.

Where multiple comparisons were made across lifestyle behavior outcomes, Bonferroni correction was applied to control for type I error. The adjusted significance threshold was set at α/N, where α=0.05 and N is the number of comparisons. With eight outcomes, this yielded an adjusted threshold of *P*<.006.

Model diagnostics were conducted to assess multicollinearity and model fit (eg, pseudo R², Akaike Information Criterion). All statistical tests were two-sided, and *P*<.05 was considered statistically significant unless otherwise corrected via Bonferroni adjustment. Missing data were handled using complete-case analysis, and follow-up rates were reported to account for potential attrition bias.

### Ethical Considerations

The study protocol was reviewed and approved by the Institutional Ethics Committee of Anusandhan Trust, Mumbai (reference number IEC26/2021) prior to implementation. The research was conducted in accordance with the ethical principles outlined in the Declaration of Helsinki and relevant national guidelines for biomedical and health research involving human participants. Informed consent was obtained from all participants before enrolment. Participants were informed about the study objectives, procedures, and potential risks and benefits and were assured that their participation was voluntary, with the right to withdraw at any time without affecting regular standard care. To ensure privacy and confidentiality, no personal identifiers were collected. The dataset used for analysis was anonymized and securely stored in password-protected files (server at Arogya World) and will be retained for 3 years, accessible only to the study investigators. This study was reported in accordance with the Transparent Reporting of Evaluations with Nonrandomized Designs (TREND) statement [17]. No monetary or material compensation was provided to participants for their participation. Additionally, no identifiable participant images or other personal visual materials were collected or included in the manuscript or appendices. In addition, regulatory guidelines of the Telecom Regulatory Authority of India (TRAI) were followed in sending text messages [18]. Our study was a quasi-experimental design and was carried out as a formative evaluation to understand how the intervention works in real settings, how participants engage with it, and how it could be improved, and there was no randomization procedure; hence, this study did not require mandatory clinical trials registration-India (CTRI) registration.

## Results

### Sociodemographic Characteristics of Participants

**Figure 2:**
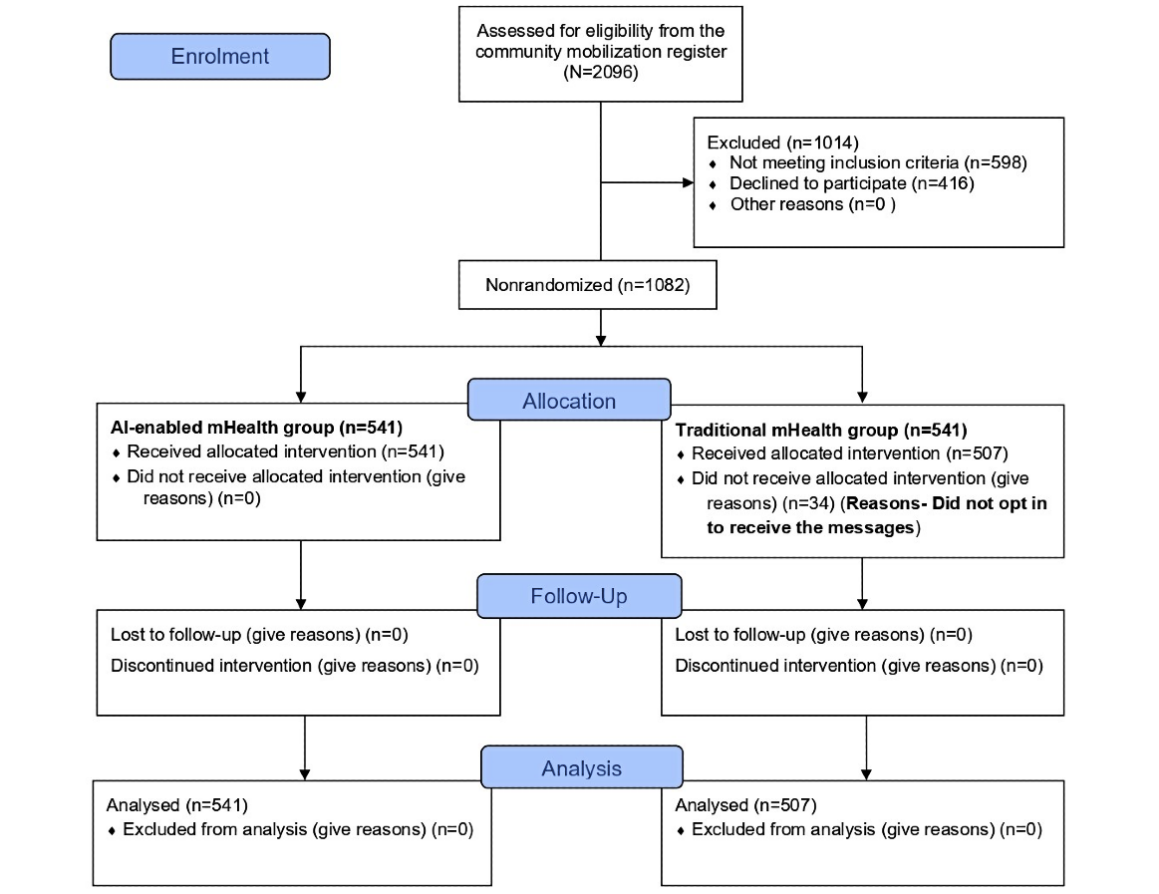
CONSORT diagram showing the flow of participants through each stage of a randomized trial. AI: artificial intelligence; CONSORT: Consolidated Standards of Reporting Trials; mHealth: mobile health.

Figure 2 shows the participant selection flow diagram. Of the 1082 participants enrolled, 1048 (96.9%) completed the 6-month follow-up and were included in the analysis (n=541, 50%, in the AI-based mHealth [intervention] group and n=507, 48.1%, in the traditional mHealth [control] group), with 387 (36.9%) males and 661 (63.1%) females. The participants were largely within the age range of 26-50 years (n=723, 69%). Educational attainment varied across the sample, with nearly half of the participants (n=517, 49.3%) having completed some level of schooling and only 73 (6.9%) holding postgraduate or higher qualifications. Employment status showed that 76% (n=796) of the participants were employed, with a slightly higher proportion of employed individuals in the intervention group (n=426, 78.6%) compared to the control group (n=371, 73.1%). Table 1 details the sociodemographic characteristics of both groups.

**Table 1:** Sociodemographic characteristics of the AI^a^-enabled (intervention) and traditional (control) mHealth^b^ groups in the rural district of Gulbarga, Karnataka, South India, 2022 (N=1048).

Characteristics			Total participants, n (%)		Control group (n=507), n (%)		Intervention group (n=541), n (%)		*P* value
**Age group (years)**									
	18-25	252 (24.0)		133 (26.2)		119 (22.0)		—^c^	
	26-35	343 (32.7)		155 (30.6)		188 (34.7)		.34	
	36-50	380 (36.3)		184 (36.3)		196 (36.2)		—	
	>50	73 (7.0)		35 (6.9)		38 (7.0)		—	
**Gender**									
	Male	387 (36.9)		180 (35.5)		207 (38.3)		.36	
	Female	661 (63.1)		327 (64.5)		334 (61.7)		—	
**Working status**									
	No	252 (24.0)		137 (26.9)		116 (21.3)		—	
	Yes	796 (76.0)		371 (73.1)		426 (78.6)		.003	
**Education**									
	Some schooling	517 (49.3)		254 (50.1)		254 (50.1)		.48	
	College or preuniversity	216 (20.6)		109 (21.5)		109 (21.5)		—	
	Professional diploma	53 (5.01)		20 (3.9)		20 (3.9)		—	
	Undergraduate degree	189 (18.0)		87 (17.2)		87 (17.2)		—	
	Postgraduate degree or higher	73 (6.9)		37 (7.3)		37 (7.3)		—	

^a^AI: artificial intelligence.

^b^mHealth: mobile health.

^c^Not applicable.

### Adherence to Different Components of Diabetes Prevention in Intervention and Control Groups

#### Physical Activity

The most notable intervention effects were observed in physical activity patterns, particularly in the intervention group. The percentage of participants engaging in regular physical activity increased from 66.7% (n=361) at baseline to 72.8% (n=394) at endline (*P*=.02) compared to the control group (n=333, 65.7%, at baseline to n=353, 69.6%, at endline; *P*=.18). Additionally, the duration of physical activity demonstrated substantial improvements in the intervention group, reporting a 15.4% increase in participants engaging in 30 minutes or more of daily exercise (*P*<.001) compared to a 14.8% increase in the control group (*P*<.001). Table 2 details the adherence of both groups to different components of diabetes prevention.

**Table 2:** Adherence to different components of diabetes prevention among the AI^a^-enabled (intervention) and traditional (control) mHealth^b^ groups in the rural district of Gulbarga, Karnataka, South India, 2022 (N=1048).

Variables			Control group (n=507)							Intervention group (n=541)					
			Baseline, n (%)	Endline, n (%)	Difference (%)		*P* value^c^		Baseline, n (%)			Endline, n (%)		Difference (%)	*P* value^c^
**Daily servings of fruits**															
	Yes	481 (94.8)		472 (93.1)	–1.7	<.001		513 (94.8)			513 (94.8)		0		.99
	No	26 (5.2)		35 (6.9)	1.8	.24		28 (5.2)			28 (5.2)		0		N/A^d^
**Daily servings of green vegetables**															
	Yes	504 (99.4)		504 (99.4)	0	.99		538(99.4)			540 (99.8)		0.4		.22
	No	3 (0.6)		3 (0.6)	0	.99		3 (0.5)			1 (0.2)		–0.3		.31
**Physical activity**															
	Currently physically active	333 (65.7)		353 (69.6)	3.9	.18		361 (66.7)			394 (72.8)		6.1		.02
	No	174 (34.3)		154 (30.4)	–3.9	.18		180 (33.3)			147 (27.2)		–6.1		.03
**Duration of doing physical activity**															
	<30 minutes	170 (33.5)		134 (26.4)	–7.1	.01		189 (34.9)			155 (28.6)		–6.3		.026
	≥30 minutes	197 (38.8)		272 (53.6)	14.8	<.001		217 (40.1)			300 (55.4)		15.4		<.001
	Do not know	140 (27.6)		101 (19.9)	–7.7	<.001		135 (24.9)			86 (15.9)		–9		<.001
**Use of stairs**															
	Yes	461 (91.0)		447 (88.1)	–2.9	.07		494 (91.3)			474 (87.6)		–3.7		.48
	No	46 (9.0)		60 (11.8)	2.7	.15		47 (8.7)			67 (12.4)		3.7		.03
**Daily chores**															
	Yes	493 (97.8)		489 (96.5)	–1.3	.37		523 (96.7)			531 (98.2)		1.5		.11
	No	14 (2.7)		18 (3.5)	0.8	.29		18 (3.3)			10 (1.8)		–1.5		.13
**Household chores**															
	Yes	474 (93.4)		497 (98.0)	4.6	.003		506 (93.5)			530 (98.0)		4.5		.009
	No	33 (6.5)		10 (2.0)	–4.6	<.001		35 (6.5)			11 (2.0)		–4.5		<.001
**Working in farms/fields- (3) Never**															
	Yes	346 (73.5)		407 (80.2)	6.7	.014		410 (75.7)			451 (83.4)		8		.002
	Never	134 (26.9)		100 (19.7)	–7.2	.01		131 (24.2)			90 (16.6)		–7.6		.002

^a^AI: artificial intelligence.

^b^mHealth: mobile health.

^c^*P* values were obtained using chi-square tests for categorical variables and independent-sample *t* tests for continuous variables. Bonferroni correction was applied for the eight behavioral outcomes presented; results with *P*<.006 were considered statistically significant.

^d^N/A: not applicable.

#### Intake of Fruits and Vegetables

Both groups exhibited high baseline adherence to daily fruit and vegetable intake, with minimal changes observed postintervention. In the intervention group, 94.8% (n=513) of the participants continued to meet the recommended servings of fruits and 99.8% (n=540) for green vegetables, showing a slight improvement from baseline for vegetables (0.4%; *P*=.22). Similarly, the control group maintained comparable levels of dietary adherence, with only a negligible decrease in fruit intake (–1.7%; *P*<.001) and no change in vegetable intake (Table 2).

#### Behavioral Changes

Among participants receiving the AI-enabled mHealth intervention, the proportion who preferred walking short distances for daily chores rose from 96.7% (n=523) to 98.2% (n=531). This represented an increase of 1.5% (95% CI –0.4 to 3.4, *P*=.11). The use of stairs declined from 91.3% (n=494) to 87.6% (n=474), a change of –3.7% (95% CI –7.3 to 0.1, *P*=.48). In the control group, walking short distances fell from 97.8% (n=493) to 96.5% (n=489), a change of –1.3% (95% CI –2.9 to 1.4, *P*=.37), and stair use declined from 91.0% (n=461) to 88.1% (n=447), a change of –2.9% (95% CI –6.7 to 0.9, *P*=.07).

Participation in household chores increased significantly in both groups (Table 2). In the intervention group, the proportion of participants engaging in household chores rose from 93.5% (n=506) to 98% (530), an absolute gain of 4.5% (95% CI 2.1-6.9, *P*=.009). The control group showed a similar improvement (from n=474, 93.4%, to n=497, 98%), which also reached significance. Agricultural work participation increased from 75.7% (n=410) to 83.4% (n=451) in the intervention group (7.7%, 95% CI 2.8-12.4, *P*=.002) and from 73.5% (n=346) to 80.2% (n=407) in the control group (6.7%, 95% CI 1.5-11.9, *P*=.014).

### Primary Outcomes

#### Physical Activity

Table 3 shows the logistic regression results for achieving at least 30 minutes of daily physical activity at endline. After adjusting for baseline status and other covariates, there was no significant difference between the two groups. Participants in the intervention group had similar odds of meeting the 30-minute daily physical activity goal compared to those in the control group (aOR 1.0, 95% CI 0.7-1.3, *P*=.74). Baseline physical activity was a strong independent predictor of endline physical activity (aOR 2.1, 95% CI 1.5-3.1, *P*<.001). Older age was associated with greater odds of regular physical activity (aOR 3.8, 95% CI 1.6-9.3 for >50 years vs 18-25 years, *P*=.003), while being employed was associated with lower odds of daily physical activity (aOR 0.2, 95% CI 0.1-0.3, *P*<.001). These findings suggest that participant characteristics, rather than intervention type, are more influential in determining physical activity outcomes.

**Table 3:** Factors associated with ≥30 minutes of physical activity at endline among AI^a^-enabled (intervention) and traditional (control) mHealth^b^ groups in rural Gulbarga, Karnataka, 2022 (N=1048).

Variable		cOR^c^ (95% CI)	*P* value	aOR^d^ (95% CI)	*P* value^e^
**Age group (years)**					
	18-25 (reference)	—^f^	—	—	—
	26-35	1.8 (1.2-2.6)	.004	2.4 (1.4-3.9)	.001
	36-50	2.3 (1.5-3.5)	<.001	3.7 (2.1-6.5)	<.001
	>50	2.6 (1.2-5.6)	.01	3.8 (1.6-9.3)	.003
**Gender**					
	Male (reference)	—	—	—	—
	Female	1.1 (0.8-1.4)	.66	—	—
**Education**					
	College or preuniversity	2.1 (1.3-3.3)	.002	3.2 (1.9-5.8)	<.001
	Undergraduate degree	0.9 (0.6-1.3)	.54	1.0 (0.6-1.5)	.95
	Postgraduate degree and higher	1.0 (0.6-1.7)	.92	1.0 (0.5-1.8)	.90
	Some schooling (reference)	—	—	—	—
	Professional diploma	1.2 (0.7-2.1)	.56	1.0 (0.-2.0)	.88
**Working status**					
	No (reference)	—	—	—	—
	Yes	0.4 (0.3-0.5)	<.001	0.2 (0.1-0.3)	<.001
**Baseline** **≥30 minutes/day of physical activity**					
	No (reference)	—	—	—	—
	Yes	1.6 (1.2-2.2)	.003	2.1 (1.5-3.1)	<.001
**Use of stairs**					
	No (reference)	—	—	—	—
	Yes	1.8 (1.1-2.8)	.014	2.6 (1.4-4.7)	.001
**Household chores**					
	No (reference)	—	—	—	—
	Yes	0.5 (0.2-1.6)	.25	—	—
**Walk down small distances for daily chores**					
	No (reference)	—	—	—	—
	Yes	0.4 (0.1-1.1)	.07	0.6 (0.1-2.2)	.41
**Farm work**					
	No (reference)	—	—	—	—
	Yes	0.6 (0.4-0.9)	.03	0.3 (0.2-0.6)	<.001
**Intervention group**					
	AI-enabled mHealth	1.0 (0.7-1.3)	.74	—	—
	Traditional mHealth (reference)	—	—	—	—

^a^AI: artificial intelligence.

^b^mHealth: mobile health.

^c^cOR: cured odds ratio.

^d^aOR: adjusted odds ratio.

^e^*P* value from adjusted analysis (multivariable logistics regression). Adjusted variables: age, education, working status, baseline physical activity, use of stairs, daily chores, and farm work.

^f^Not applicable.

#### Daily Fruit Intake

As shown in Table 4, there was no significant difference in daily fruit consumption between the two groups at endline. After adjusting for baseline fruit intake and covariates, the odds of consuming fruit daily were modestly higher in the intervention group (aOR 1.4, 95% CI 0.8-2.3), though not statistically significant (*P*=.24). Baseline fruit intake was strongly predictive of endline fruit intake (*P*<.001). Age and employment were also associated with daily fruit intake: participants aged 26-35 years had higher odds of eating fruit daily than those aged 18-25 years (aOR 4.7, 95% CI 1.9-11.8, *P*=.001), while being employed was linked to lower odds of daily fruit intake (aOR 0.3, 95% CI 0.1-0.8, *P*=.02).

**Table 4:** Factors associated with daily fruit intake at endline among AI^a^-enabled (intervention) and traditional (control) mHealth^b^ groups in rural Gulbarga, Karnataka, 2022 (N=1048).

Variable		cOR^c^ (95% CI)	*P* value	aOR^d^ (95% CI)	*P* value^e^
**Age group (years)**					
	18-25 (reference)	—^f^	—	—	—
	26-35	1.8 (0.9-3.8)	0.114	4.7 (1.9-11.8)	0.001
	36-50	0.9 (0.5-1.8)	0.854	3.4 (1.4-8.5)	0.007
	>50	1.2 (0.3-4.4)	0.778	3.7 (0.8-16.9)	0.091
**Gender**					
	Male (reference)	—	—	—	—
	Female	1.4 (0.8-2.3)	0.204	—	—
**Education**					
	College or preuniversity	1.7 (0.8-3.4)	0.152	1.3 (0.6-3.1)	0.510
	Undergraduate degree	7.4 (2.3-24.0)	0.001	4.4 (1.2-15.9)	0.025
	Postgraduate degree and higher	9.1 (1.2-66.9)	0.030	4.5 (0.6-34.9)	0.151
	Some schooling (reference)	—	—	—	—
	Professional diploma	1.6 (0.6-4.7)	0.356	0.7 (0.2-2.2)	0.551
**Working status**					
	No (reference)	—	—	—	—
	Yes	0.2 (0.1-0.5)	0.001	0.3 (0.1-0.8)	0.022
**Baseline fruit intake**					
	No (reference)	—	—	—	—
	Yes	36.4 (19.2-68.9)	<0.001		<0.001
**Physical activity**					
	No (reference)	—	—	—	—
	Yes	1.1 (0.6-1.9)	0.795	—	—
**Use of stairs**					
	No (reference)	—	—	—	—
	Yes	5.7 (3.3-9.8)	0.001	3.5 (1.7-7.3)	0.001
**Household chores**					
	No (reference)	—	—	—	—
	Yes	—	—	—	—
**Walk down small distances for daily chores**					
	No (reference)	—	—	—	—
	Yes	—	—	—	—
**Farm work**					
	No (reference)	—	—	—	—
	Yes	1.1 (0.6-2.0)	0.845	—	—
**Intervention group**					
	AI-enabled mHealth	1.4 (0.8-2.3)	0.241	—	—
	Traditional mHealth (reference)	—	—	—	—

^a^AI: artificial intelligence.

^b^mHealth: mobile health.

^c^cOR: cured odds ratio.

^d^aOR: adjusted odds ratio.

^e^*P* value from adjusted analysis (multivariable logistics regression). Adjusted variables: age, education, working status, baseline fruit intake, physical activity, use of stairs, and household chores.

^f^Not applicable.

#### Body Mass Index

ANCOVA (Table 5) showed no significant difference in the mean BMI between the two groups at endline. After adjusting for baseline BMI, the mean difference was essentially 0 (–0.0 kg/m², 95% CI –0.6 to 0.5, *P*=.95). Baseline BMI was a strong determinant of endline BMI (*P*<.001), but no intervention effect was detected.

**Table 5:** Factors associated with the BMI at endline among AI^a^-enabled (intervention) and traditional (control) mHealth^b^ groups in rural Gulbarga, Karnataka, 2022 (N=1048).

Variable		Coefficient (95% CI)	SE	*P* value^c^
**Age group (years)**				
	18-25 (reference)	—^d^	—	—
	26-35	1.7 (0.9 to 2.6)	0.42	<.001
	36-50	2.1 (1.2 to 3.0)	0.45	<.001
	>50	3.2 (1.7 to 4.7)	0.76	<.001
**Gender**				
	Male (reference)	—	—	—
	Female	–0.6 (–1.2 to 0.001)	0.03	.05
**Education**				
	College or preuniversity	0.3 (–0.5 to 1.2)	0.40	.41
	Undergraduate degree	0.7 (–0.1 to 1.5)	0.40	.10
	Postgraduate degree and higher	0.5 (–0.6 to 1.6)	0.50	.37
	Some schooling (reference)	—	—	—
	Professional diploma	1.6 (0.3 to 2.8)	0.60	.014
**Working status**				
	No (reference)	—	—	—
	Yes	0.3 (–0.4 to 1.0)	0.40	.41
**Daily servings of fruits**				
	No (reference)	—	—	—
	Yes	0.6 (–0.6 to 1.8)	1.00	.33
**Daily servings of green vegetables**				
	No (reference)	—	—	—
	Yes	–0.5 (–5.0 to 4.1)	–0.20	.84
BMI (baseline)		0.4 (0.4 to 0.5)	16.10	<.001
**Physical activity**				
	No (reference)	—	—	—
	Yes	–0.6 (–1.2 to 0.1)	–1.70	.09
**Use of stairs**				
	No (reference)	—	—	—
	Yes	0.8 (–0.2 to 1.7)	1.60	.11
**Household chores**				
	No (reference)	—	—	—
	Yes	1.2 (–0.8 to 3.2)	1.20	.25
**Farm work**				
	No (reference)	—	—	—
	Yes	0.7 (–0.0 to 1.5)	0.40	.06
**Intervention group**				
	AI-enabled mHealth	–0.0 (–0.6 to 0.5)	0.30	.95
	Traditional mHealth (reference)	—	—	—

^a^AI: artificial intelligence.

^b^mHealth: mobile health.

^c^ANCOVA.

^d^Not applicable.

### Exploratory Behavioral Outcomes

Other incidental physical activity measures also showed no significant differences between the two groups. The aORs (95% CI) for the intervention versus the control group were as follows: stair use (aOR 0.9, 95% CI 0.7-1.4, *P*=.79), walking for chores (aOR 2.4, 95% CI 1.0-6.1, *P*=.06), helping with household chores (aOR 1.0, 95% CI 0.4-2.3, *P*=.94), and farm work (aOR 1.3, 95% CI 0.9-1.8, *P*=.19). See Tables S1-S4 in Multimedia Appendix 2.

### Message Delivery Rate, Responses to Feedback, and Engagement Over 6 months

Across both study arms, 85,000 WhatsApp messages were scheduled. About 82,600 (97.2%) of these were successfully delivered (95% CI 96.9-97.1), and approximately 77,000 (93.2%) of these were marked as read (95% CI 92.8-93.2) .At baseline, 65.1% (352) of intervention group participants reported making changes in response to messages; at endline, this proportion was 66.4% (n=359 participants). In comparison, 64.7% (n=328) of participants in the control group at both baseline and endline reported making changes in response to messages; see Table S5 in Multimedia Appendix 2. In addition, engagement remained relatively stable over the 6‐month period (Table 2).

## Discussion

### Principal Findings

This study evaluated the effectiveness of an AI-enabled mHealth intervention (mDiabetes) versus traditional mHealth for promoting diabetes prevention behaviors in rural India. Our primary finding was that there were no significant differences between the AI-enabled and traditional mHealth groups for the primary outcomes of physical activity and dietary behaviors after 6 months of intervention. Both interventions demonstrated effectiveness in maintaining and promoting physical activity behaviors, with baseline activity being the strongest predictor (aOR 2.1, 95% CI 1.5-3.1, *P*<.001). This suggests that any form of consistent mHealth messaging may be beneficial for diabetes prevention regardless of AI customization. The finding that age>50 years is associated with higher odds of physical activity (aOR 3.8, 95% CI 1.6-9.3, *P*=.003) is particularly encouraging, as this demographic faces higher diabetes risk. Conversely, employment being associated with lower physical activity odds (aOR 0.2, 95% CI 0.1-0.3, *P*<.001) highlights the real-world barriers that working adults face in rural settings. Our finding are consistent with recent reviews that show that although mHealth interventions generally improve physical activity within groups, the incremental benefit of AI- or app-based enhancements over active comparators is often modest [19,20].

Our findings are in contrast with those of existing studies, which generally report declining physical activity with advancing age due to reduced mobility, chronic conditions, and competing health limitations [21]. However, some studies have reported that older adults may engage more consistently in routine or incidental activities, such as walking, household chores, or agricultural work, particularly in rural areas [22].

For daily fruit intake, although the intervention effect was not significant, the age-specific patterns are noteworthy. Participants aged 26-35 years showed higher odds of daily fruit intake (aOR 4.7, 95% CI 1.9-11.8, *P*=.001), suggesting this age group may be more receptive to dietary behavior change. The negative association with employment (aOR 0.3, 95% CI 0.1-0.8, *P*=.022) again underscores socioeconomic barriers to healthy behaviors. Similar findings have been reported in other mHealth interventions in India, where improvements in fruit intake were observed but often without strong between-group differences. For instance, the *SMART Eating* trial in Chandigarh documented a significant rise in fruit consumption using IT-enabled strategies compared to standard education [23]. These findings suggest that AI-enabled approaches may promote healthier choices, such as fruit consumption [23].

To the best of our knowledge, this study is the first in India to rigorously, directly compare, in a large rural cohort, an AI-enabled mHealth intervention (dynamic) with a traditional static mHealth intervention for diabetes prevention. Key novel features include its real-world rural setting with minimal exclusions, the innovative use of reinforcement learning AI to customize messages based on individual feedback, and its focus on adults without diabetes for prevention rather than diabetes management [14]. Additionally, the study achieved a high retention rate of 97%, demonstrating both feasibility and acceptability of mHealth in rural populations.

ANCOVA revealed no significant difference in the mean BMI between the two groups at endline after adjusting for the baseline BMI and other covariates (mean difference –0.0 kg/m², 95% CI –0.6 to 0.5, *P*=.95). Previous research suggests that even noncustomized mHealth interventions can yield positive outcomes, particularly in contexts where strong community support structures exist. Such interventions, regardless of personalization, have the potential to influence health behaviors; however, their effectiveness may be enhanced when integrated with complementary support mechanisms [24,25]. A recently published study [26] showed that combined mHealth and community health education intervention improves diabetes awareness and healthy habits in rural areas, indicating potential for lasting outcomes and guiding future public health efforts in rural settings [26].

The implications of these findings for public health practice are substantial. The demonstrated effectiveness of the AI-based mHealth intervention in increasing physical activity and maintaining healthy behaviors suggests that such tools could be crucial in diabetes prevention programs, especially in rural and underserved areas where health care resources are limited. Moreover, the ability of AI-driven interventions to provide customized guidance and real-time feedback makes them particularly suited for scalable, population-level health initiatives. These mHealth interventions can bridge significant gaps in health care delivery, particularly in resource-constrained settings [26]. However, the findings also suggest that a “one-size-fits-all” approach may not be sufficient. Integrating AI-driven mHealth interventions with existing health care systems, including community health workers and primary care providers, could enhance their effectiveness and sustainability.

Additionally, the existing literature also emphasizes the importance of physical activity and active lifestyles in managing health outcomes, particularly in mHealth interventions. Several studies have demonstrated that regular physical activity, such as engaging in 30 minutes or more of exercise daily, significantly reduces the risk of chronic conditions, including diabetes and cardiovascular diseases [27,28]. Moreover, nonexercise activities, such as household chores and farm work, which showed significant associations with better health outcomes in this study, are well recognized as beneficial contributors to physical and metabolic health. Evidence suggests that active engagement in household tasks and manual labor can improve cardiovascular health and reduce the risk of complications in chronic diseases like diabetes [29,30].

The significance of using stairs and walking for daily chores, particularly in the mHealth AI group, mirrors findings from other studies that promote incidental physical activity as a valuable component of overall health management [31]. Activities like stair climbing, which are simple to incorporate into daily routines, have been shown to improve cardiovascular function and aid in glucose regulation, both critical factors in diabetes prevention and management [32]. These findings underscore the importance of integrating physical activity, both structured and unstructured, into health interventions to enhance their effectiveness, particularly in AI-driven mHealth programs.

### Strengths and Limitations

This study marks the first national attempt to use WhatsApp-based text messaging on mobile phones to support educational interventions aimed at preventing diabetes. The strengths of this study include a large sample size (N=1048), which offers adequate statistical power to detect intervention effects, and a high retention rate of 97% that minimizes selection bias and enhances the reliability of findings. Conducting the study in a real-world rural community setting further strengthened external validity. Additionally, rigorous statistical approaches were used, including appropriate adjustments for multiple comparisons, and a comprehensive set of outcome measures covered both behavioral changes and knowledge gains [14]. Moreover, in-person data collection by trained FLWs helped ensure data quality and reduced the potential for response bias, and the development of the intervention was guided by the transtheoretical model of behavior change, ensuring a solid theoretical basis.

However, the study has certain limitations. First, the reliance on self-reported data for physical activity and dietary habits (primary outcomes) is subject to recall and social desirability biases, potentially overestimating the true effects of the interventions. Second, the relatively short intervention period of 6 months limits the assessment of long-term sustainability of behavior changes. Third, the recruitment process involved an opt-in procedure, which could introduce selection bias, as participants who chose to participate may be more motivated to adopt healthy behaviors than the general population. Finally, biochemical markers were not objectively assessed to evaluate the clinical outcomes due to a lack of financial resources, which would have provided more detailed insights into the biological effects of the intervention. To address these limitations, further studies could incorporate objective measures of primary outcomes, such as accelerometers or pedometers for physical activity and validated dietary assessment tools, including food frequency questionnaires or 24-hour dietary recalls. Extending the intervention and follow-up periods would allow for evaluation of the sustainability of behavior changes over time. Recruitment strategies that ensure a more representative sample of the target population could help minimize selection bias. Furthermore, the inclusion of biochemical markers or other clinical endpoints would provide more robust evidence of the physiological and metabolic impacts of the intervention, enhancing the translational relevance of the findings. Additionally, reliance on WhatsApp messaging may have excluded individuals without smartphone access, limiting generalizability to economically disadvantaged populations. Collectively, these limitations likely bias the results toward the null hypothesis, suggesting that the true effects of the interventions may be underestimated rather than exaggerated.

### Conclusion

This study revealed that engaging, well-designed static messages can be just as effective as complex AI-personalized approaches in diabetes prevention, challenging prevailing assumptions and pointing to cost-effective, scalable options for program managers and policymakers.

This study demonstrates that traditional mHealth interventions are as effective as AI-enabled approaches for promoting diabetes prevention behaviors in rural India. Although this finding challenges assumptions about the superior effectiveness of AI-powered health interventions, it provides valuable evidence for scalable, cost-effective diabetes prevention strategies. The high acceptability and retention rates of both AI-driven and traditional interventions suggest that consistent health messaging through accessible platforms like WhatsApp can effectively support diabetes prevention efforts in rural populations.

Rather than viewing the lack of AI superiority as a negative finding, this result should be interpreted as evidence for the democratization of effective health interventions. Simple, well-designed mHealth programs can achieve meaningful health behavior changes without requiring sophisticated technological infrastructure, making diabetes prevention more accessible to underserved rural populations.

## Appendix

Multimedia Appendix 1 Dataset used for analysis.

Multimedia Appendix 2 Factors associated with stairs use at endline; walking for chores at endline; helping in household chores at endline; farm work at endline; and message delivery rate, responses to feedback, and engagement over 6 months in artificial intelligence (AI)–enabled and traditional mobile health (mHealth) groups in rural Gulbarga, Karnataka, 2022 (N=1048).

## Abbreviations

AI: artificial intelligence
aOR: adjusted odds ratio
cOR: cured odds ratio
DQN: Deep Q-Learning
FLW: frontline worker
mHealth: mobile health

## Data Availability

The data supporting the findings of this study are available within the manuscript and have been uploaded as Multimedia Appendix 1.
