# Supplementary material for: Artificial Intelligence–Enabled Mobile Health Intervention (mDiabetes) to Reduce Diabetes Risk Behaviors in Rural India: Quasi-Experimental Pre-Post Study
Source: J Med Internet Res. 2025 Dec 5;27:e79283. doi: 10.2196/79283 (PMC12717508; doi:10.2196/79283)
Supplement: Multimedia Appendix 2 [file jmir_v27i1e79283_app2.docx]

**Table S1. Factors associated with stairs use at endline among AI-enabled and traditional mHealth groups in rural Gulbarga, Karnataka, 2022 (N = 1,048).**

| **Variable** | **COR (95% CI)** | **P-value** | **aOR (95% CI)** | **P-value*** |
| --- | --- | --- | --- | --- |
| **Age group (years)** | | | | |
| 18-25 | ref |  | ref |  |
| 26-35 | 0.4 (0.2 – 0.9) | 0.031 | 0.6 (0.2 – 1.5) | 0.278 |
| 36-50 | 0.2 (0.1 – 0.4) | <0.001 | 0.3 (0.1 – 0.8) | 0.017 |
| >50 | 0.1 (0.1 – 0.4) | <0.001 | 0.3 (0.1 – 1.0) | 0.051 |
| **Gender** | | | | |
| Male | ref |  | ref |  |
| Female | 0.2 (0.1 – 0.3) | <0.001 | 0.1 (0.1 – 2.4) | <0.001 |
| **Education** | | | | |
| College or preuniversity | 2.8 (1.6 – 4.9) | 0.001 | 2.0 (1.0 – 3.8) | 0.037 |
| Undergraduate | 7.9 (3.6 – 17.3) | <0.001 | 4.4 (1.9 – 10.1) | <0.001 |
| Postgraduate and above | 5.6 (2.0 _15.6) | 0.001 | 4.1 (1.3 – 12.7) | 0.015 |
| Some schooling | ref |  | ref |  |
| Prof diplomas | 17.4 (2.4 – 127.1) | 0.005 | 24.6 (3.3 – 184.6) | 0.002 |
| **Working status** | | | | |
| No | ref |  | ref |  |
| Yes | 0.4 (0.2 – 0.7) | <0.001 | 0.3 (0.1 – 0.5) | <0.001 |
| **Baseline stairs use** | | | | |
| No | ref |  | ref |  |
| Yes | 0.8 (0.4 – 1.6) | 0.451 | 0.4 (0.1 – 0.9) | 0.024 |
| **Physical activity** | | | | |
| No | ref |  | ref |  |
| Yes | 0.7 (0.4 – 1.1) | 0.078 | 0.8 (0.5 – 1.4) | 0.430 |
| **Household chores** | | | | |
| No | ref |  | ref |  |
| Yes | 1.7 (0.6 – 5.2) | 0.331 | - | - |
| **Walk down small distances for daily chores** | | | | |
| No | ref |  |  |  |
| Yes | 10.9 (5.1 – 23.6) | <0.001 | 32.9 (11.6 – 93.5) | <0.001 |
| **Farm work** | | | | |
| No | ref |  | ref |  |
| Yes | 1.1 (0.7 – 1.8) | 0.628 | - | - |
| **Intervention group** | | | | |
| AI-enabled mHealth | 0.9 (0.7 – 1.4) | 0.785 |  |  |
| Traditional mHealth | ref | ref | - | - |

*COR- Cured Odds Rattio, aOR- adjusted Odds Ratio, *P-value from adjusted analysis (Multivariable logistics regression). Adjusted variables- age, gender, education, working status, baseline stairs use, physical activity, and daily chores.*

**Table S2. Factors associated with walking for chores** **at endline among AI-enabled and traditional mHealth groups in rural Gulbarga, Karnataka, 2022 (N = 1,048).**

| **Variable** | **COR (95% CI)** | **P-value** | **aOR (95% CI)** | **P-value*** |
| --- | --- | --- | --- | --- |
| **Age group (years)** | | | | |
| 18-25 | ref |  | ref |  |
| 26-35 | 1.0 (0.3 – 3.4) | 0.996 | 1.2 (0.3 – 4.7) | 0.827 |
| 36-50 | 0.7 (0.2 – 2.3) | 0.573 | 0.8 (0.2 – 3.1) | 0.722 |
| >50 | 0.4 (0.1 – 1.6) | 0.181 | 0.4 (0.1 – 2.5) | 0.347 |
| **Gender** | | | | |
| Male | ref |  | ref |  |
| Female | 0.9 (0.4 – 2.1) | 0.893 | - | - |
| **Education** | | | | |
| College or preuniversity | 1.2 (0.4 – 3.4) | 0.690 | - | - |
| Undergraduate | - | - | - | - |
| Postgraduate and above | 0.8 (0.3 – 2.4) | 0.665 | - | - |
| Some schooling | ref |  | ref |  |
| Prof diplomas | 1.2 (0.3 – 5.3) | 0.807 | - | - |
| **Working status** | | | | |
| No | ref |  | ref |  |
| Yes | 2.9 (1.4 – 6.3) | 0.005 | 3.4 (0.4 – 8.5) | 0.009 |
| **Baseline walking for chores** | | | | |
| No | ref |  | ref |  |
| Yes | 8.0 (2.8 – 22.6) | <0.001 | 4.0 (1.0 – 15.5) | 0.043 |
| **Physical activity** | | | | |
| No | ref |  | ref |  |
| Yes | 1.4 (0.6 – 3.1) | 0.409 | - | - |
| **Uses stairs** | | | | |
| No | ref |  | ref |  |
| Yes | 10.9 (5.0 – 23.6) | <0.001 | 12.2 (4.9 – 30.6) | <0.001 |
| **Household chores** | | | | |
| No | ref |  | ref |  |
| Yes | 18.3 (6.5- 51.5) | <0.001 | 17.0 (4.4 – 65.8) | <0.001 |
| **Farm work** | | | | |
| No | ref |  | ref |  |
| Yes | 4.8 (2.2 – 10.2) | <0.001 | 3.9 (1.6 – 9.6) | 0.003 |
| **Intervention group** | | | | |
| AI-enabled mHealth | 1.9 (0.9 – 4.3) | 0.093 | 2.4 (1.0 – 6.1) | 0.056 |
| Traditional mHealth | ref |  | ref |  |

*COR- Cured Odds Rattio, aOR- adjusted Odds Ratio, *P-value from adjusted analysis (Multivariable logistics regression). Adjusted variables- age, working status, baseline walking for chores, uses stairs, household chores, farm work, and intervention group.*

**Table S3. Factors associated with Helping household chores at endline among AI-enabled and traditional mHealth groups in rural Gulbarga, Karnataka, 2022 (N = 1,048).**

| **Variable** | **COR (95% CI)** | **P-value** | **aOR (95% CI)** | **P-value*** |
| --- | --- | --- | --- | --- |
| **Age group (years)** | | | | |
| 18-25 | ref |  | ref |  |
| 26-35 | 0.8 (0.3 – 2.7) | 0.761 | - | - |
| 36-50 | 2.2 (0.5 – 8.9) | 0.268 | - | - |
| >50 | 0.5 (0.1 – 3.0) | 0.483 | - | - |
| **Gender** | | | | |
| Male | ref |  | ref |  |
| Female | 0.9 (0.3 – 2.1) | 0.731 | - | - |
| **Education** | | | | |
| College or preuniversity | 0.9 (0.3 – 2.9) | 0.854 | - | - |
| Undergraduate | 1.5 (0.4 – 5.6) | 0.527 | - | - |
| Postgraduate and above | 0.6 (0.2 – 2.3) | 0.461 | - | - |
| Some schooling | ref |  | ref |  |
| Prof diplomas | 1.4 (0.2 – 11.2) | 0.742 |  |  |
| **Working status** | | | | |
| No | ref |  | ref |  |
| Yes | 3.4 (1.4 – 8.2) | 0.006 | 3.6 (1.4 – 9.2) | 0.008 |
| **Baseline household chores** | | | | |
| No | ref |  | ref |  |
| Yes | 0.7 (0.1 – 5.4) | 0.747 | 0.7 (0.1 – 5.7) | 0.721 |
| **Physical activity** | | | | |
| No | ref |  |  |  |
| Yes | 0.4 (0.1 – 1.4) | 0.153 | 0.2 (0.1 – 0.9) | 0.034 |
| **Uses stairs** | | | | |
| No | ref |  | ref |  |
| Yes | 1.7 (0.6 – 5.2) | 0.331 | - | - |
| **Walk down small distances for daily chores** | | | | |
| No | ref |  | ref |  |
| Yes | 18.2 (6.5 – 51.5) | <0.001 | 16.3 (5.3 – 49.4) | <0.001 |
| **Farm work** | | | | |
| No | ref |  | ref |  |
| Yes | 1.8 (0.7 – 4.8) | 0.216 | - | - |
| **Intervention group** | | | | |
| AI-enabled mHealth | 1.0 (0.4 – 2.3) | 0.944 | - | - |
| Traditional mHealth | ref |  | - | - |

*COR- Cured Odds Rattio, aOR- adjusted Odds Ratio, *P-value from adjusted analysis (Multivariable logistics regression). Adjusted variables- working status, baseline household chores, physical activity, uses stairs, daily chores, farm work, and intervention group.*

**Table S4. Factors associated with farm work at endline among AI-enabled and traditional mHealth groups in rural Gulbarga, Karnataka, 2022 (N = 1,048).**

| **Variable** | **COR (95% CI)** | **P-value** | **aOR (95% CI)** | **P-value*** |
| --- | --- | --- | --- | --- |
| **Age group (years)** | | | | |
| 18-25 | ref |  | ref |  |
| 26-35 | 1.1 (0.7 – 1.7) | 0.566 | - | - |
| 36-50 | 1.3 (0.8 – 2.0) | 0.286 | - | - |
| >50 | 1.0 (0.5 – 2.2) | 0.959 | - | - |
| **Gender** | | | | |
| Male | ref |  | ref |  |
| Female | 1.1 (0.8 – 1.6) | 0.422 | - | - |
| **Education** | | | | |
| College or preuniversity | 0.5 (0.3 – 0.7) | 0.001 | 0.4 (0.3 – 0.7) | 0.002 |
| Undergraduate | 0.7 (0.5 – 1.2) | 0.199 | 0.6 (0.4 – 1.0) | 0.040 |
| Postgraduate and above | 0.2 (0.1 – 0.4) | <0.001 | 0.2 (0.1 – 0.4) | <0.001 |
| Some schooling | ref |  | ref |  |
| Prof diplomas | 0.1 (0.1 – 0.2) | <0.001 | 0.1 (0.1 – 0.3) | <0.001 |
| **Working status** | | | | |
| No | ref |  | ref |  |
| Yes | 0.8 (0.5 – 1.1) | 0.180 | 0.6 (0.4 – 0.9) | 0.028 |
| **Baseline farm work** | | | | |
| No | ref |  | ref |  |
| Yes | 6.8 (4.8 – 9.6) | <0.001 | 5.4 (3.8 – 7.8) | <0.001 |
| **Physical activity** | | | | |
| No | ref |  |  |  |
| Yes | 1.5 (1.1 – 2.1) | 0.011 | 2.1 (1.3 – 3.1) | <0.001 |
| **Uses stairs** | | | | |
| No | ref |  | ref |  |
| Yes | 1.1 (0.7 – 1.8) | 0.628 | - | - |
| **Household chores** | | | | |
| No | ref |  |  |  |
| Yes | 1.8 (0.7 – 4.8) | 0.216 | - | - |
| **Walk down small distances for daily chores** | | | | |
| No | ref |  | ref |  |
| Yes | 4.8 (2.2 – 10.2) | <0.001 | 3.5 (1.5 – 8.5) | 0.005 |
| **Intervention group** | | | | |
| AI-enabled mHealth | 1.2 (0.9 – 1.7) | 0.195 | 1.3 (0.9 – 1.8) | 0.188 |
| Traditional mHealth | ref |  |  |  |

*COR- Cured Odds Rattio, aOR- adjusted Odds Ratio, *P-value from adjusted analysis (Multivariable logistics regression). Adjusted variables- education, working status, baseline farm work, physical activity, daily chores, farm work, and intervention group.*

**Table S5. Message delivery rate, responses to feedback and engagement over six months among AI-enabled and traditional mHealth groups in rural Gulbarga, Karnataka, 2022 (N = 1,048).**

| **Variable** | **AI-enabled mHealth (n=541)** | **Traditional mHealth (n=507)** | **Total (N=1048)** |
| --- | --- | --- | --- |
| **Response to the feedback** |  |  |  |
| Yes | 501 (92.6) | 464 (91.5) | 965 (92.1) |
| No | 40 (7.4) | 43 (8.5) | 83 (8.0) |
| **Changes due to intervention** |  |  |  |
| Yes | 359 (66.4) | 328 (64.7) | 687 (65.5) |
| No | 53 (9.8) | 38 (7.5) | 91 (8.7) |
| Don’t know | 129 (23.8) | 141 (27.8) | 270 (25.8) |
